# *In vitro* and *in vivo* modeling of lipid bioaccessibility and digestion from almond muffins: The importance of the cell-wall barrier mechanism

**DOI:** 10.1016/j.jff.2017.07.046

**Published:** 2017-10

**Authors:** Terri Grassby, Giuseppina Mandalari, Myriam M.-L. Grundy, Cathrina H. Edwards, Carlo Bisignano, Domenico Trombetta, Antonella Smeriglio, Simona Chessa, Shuvra Ray, Jeremy Sanderson, Sarah E. Berry, Peter R. Ellis, Keith W. Waldron

**Affiliations:** a Biopolymers Group, Diabetes and Nutritional Sciences Division, King’s College London, Franklin-Wilkins Building, 150 Stamford Street, London SE1 9NH, UK; b Quadram Institute Bioscience, Norwich NR4 7UA, UK; c Department of Chemical, Biological, Pharmaceutical and Environmental Science, University of Messina, Viale SS. Annunziata, 98168 Messina, Italy; d Department of Biomedical, Dental, Morphological and Functional Images Sciences, University of Messina, Via C. Valeria, 98125 Messina, Italy; e Department of Gastroenterology, Guy's and St. Thomas' NHS Foundation Trust, London SE1 9RT, UK

**Keywords:** Almonds, Bioaccessibility, Ileostomy, *In vitro* digestion, Particle size

## Abstract

This study compares *in vitro* and *in vivo* models of lipid digestion from almond particles within a complex food matrix (muffins) investigating whether the cell-wall barrier regulates the bioaccessibility of nutrients within this matrix. Muffins containing small (AF) or large (AP) particles of almond were digested in triplicate using an *in vitro* dynamic gastric model (DGM, 1 h) followed by a static duodenal digestion (8 h). AF muffins had 97.1 ± 1.7% of their lipid digested, whereas AP muffins had 57.6 ± 1.1% digested. *In vivo* digestion of these muffins by an ileostomy volunteer (0–10 h) gave similar results with 96.5% and 56.5% lipid digested, respectively. The AF muffins produced a higher postprandial triacylglycerol iAUC response (by 61%) than the AP muffins. Microstructural analysis showed that some lipid remained encapsulated within the plant tissue throughout digestion. The cell-wall barrier mechanism is the main factor in regulating lipid bioaccessibility from almond particles.

## Highlights

• We investigated the mechanisms of lipid bioaccessibility from almond muffins.
• An *in vitro* dynamic gastric model was used to simulate human digestion.
• A pilot ileostomy study was performed to define the rate of lipid release.
• Microstructural analysis proved that some lipid remained encapsulated within matrix.
• The cell-wall is the main factor regulating the lipid bioaccessibility.

## 1 Introduction

It is well established that intact cell walls play an important role in regulating the bioaccessibility of intracellular lipid and other nutrients in almond seeds (Ellis et al., 2004, Mandalari et al., 2008). In this paper, we refer to bioaccessibility as the proportion of a nutrient or phytochemical compound ‘released’ from a complex food matrix during digestion and, therefore, potentially available for absorption in the gastrointestinal (GI) tract.

Despite the high lipid content of almonds (typically in the range of 50–55%), a meta-analysis of randomized controlled trials indicates that their consumption does not result in increased body weight or BMI (Flores-Mateo, Rojas-Rueda, Basora, Ros, & Salas-Salvadó, 2013), and this is believed to be, in part, due to their low lipid bioaccessibility. Novotny et al. estimated that only 76% of the energy contained within almonds (based on the Atwater factors) is actually metabolized (Novotny, Gebauer, & Baer, 2012). Accumulating evidence suggests that the limited bioaccessibility of lipids within intact almond tissue can be attributed to the structural integrity of almond cell walls, which protect encapsulated lipids from digestion during passage through the GI tract. We have demonstrated that mastication of whole natural almonds released only a small proportion (7.8%) of the total lipid (Mandalari et al., 2014). However, the overall release/digestion of lipid increased during subsequent simulated gastric (16.4%) and duodenal (32.2%) digestion, with 67.8% of the lipid remaining undigested (Mandalari et al., 2014). The lipid release from masticated almonds was in close agreement with that predicted by a theoretical model for almond lipid bioaccessibility, which had particle size and cell diameter as variables (Grassby et al., 2014, Grundy, Grassby et al., 2015). We recently demonstrated that a decrease in almond particle size (i.e. proportion of intact cells) resulted in an increased rate and extent of lipolysis in an *in vitro* model of duodenal digestion (Grundy, Wilde, Butterworth, Gray, & Ellis, 2015). All these studies provide convincing evidence that the preservation of the structural integrity of almonds during digestive transit is the major factor responsible for the limited lipid bioaccessibility of almonds. In addition to effects on energy balance, this may influence chronic and postprandial metabolic factors relating to cardio metabolic disease risk factors (Estruch et al., 2013). Indeed, a randomized crossover dietary-intervention study by Berry et al. (2008) showed that ingestion of muffins containing almond macroparticles (1.7–3.4 mm, in which ∼95% of the lipid was encapsulated by cell walls) resulted in lower postprandial lipemia (an independent risk factor for cardiovascular disease (CVD)) (Bansal et al., 2007, Lindman et al., 2010, Nordestgaard et al., 2007), compared with a nutritionally-matched muffin containing defatted almond flour and extracted free lipid (i.e. no lipid encapsulation). However, muffins containing defatted almond flour are unrealistic, so we have used muffins containing almond flour and almond particles in this study. Muffins represent a good processed food model, as they contain moisture, lipid, protein and carbohydrates, which may interact with the almonds during processing, and their production involves a number of commonly occurring processes.

The aim of the present study was to assess and compare lipid bioaccessibility from test meals containing almonds of different particle sizes (degrees of lipid encapsulation) in a dynamic *in vitro* digestion model and post-digestion in an ileostomy volunteer. This comparison is important for the validation of the dynamic gastric model (DGM) of digestion in determining its usefulness to assess the digestibility of nutrients within complex food matrices. To the best of our knowledge, this is the first investigation directly comparing the digestion of meals with a complex matrix in both the DGM and *in vivo*.

## 2 Materials and methods

### 2.1 Test meals

The test meals consisted of an almond muffin (220 g) served with custard (80 g, Bird’s Low Fat Instant Custard; Premier Ambient Products, Lincolnshire, UK). Natural (raw) almond kernels or seeds (*Amygdalus communis* L.; variety Nonpareil) were produced by Hughson Nut Inc. (Hughson, CA 95326, USA) and supplied by the Almond Board of California (Modesto, CA 95354, USA). Almonds were ground in a coffee grinder (Lloytron PLC, Lancashire, UK) and sieved to produce almond macroparticles (AP, particle size range 1700–2000 µm) and almond flour (AF, particle size <450 µm). The predicted lipid bioaccessibility values of the AF and AP were 49% and 6%, respectively (Grassby et al., 2014). The muffins were prepared from the ingredients listed in Table 1, with each muffin containing 85 g of almond as either AF or AP. In brief, the dry ingredients were thoroughly mixed by sifting them together twice, and then the wet ingredients were combined in a separate bowl, to which the dry mixture was gently incorporated. The muffin mixture was baked (using a domestic, fan-assisted oven) in muffin cases at 180 °C for 20 min, followed by 13 min at 200 °C, with the muffins protected by foil for the final 3 min. Muffins were baked in a single batch, cooled to ambient temperature, and then frozen until needed (−20 °C). The test meals included custard (prepared using the manufacturer’s instructions) to encourage the volunteers to swallow without significant mastication in order to minimize further particle size reduction. Both muffins had identical nutrient contents as calculated from the ingredients in Table 1 and nutrition tables (Food Standards Agency, 2002), except lipid which was measured by Soxhlet analysis (hexane): 48 g of lipid, 25 g of protein, 79 g of available carbohydrate (starch and sugars) and 10 g of dietary fiber. The total energy content of each muffin was 742 kcal (3161 kJ). The nutritional contribution of 80 g of custard was 1.4 g of fat, 0.5 g of protein, 9.5 g of carbohydrate and 0.1 g of dietary fiber.

**Table 1:** Ingredients for each almond muffin.

Ingredients	Weight (g)
Cornflour	10.6
Wheat flour (white, plain)	25.0
Sugar (white)	32.6
Baking powder	2.3
Skimmed milk	54.6
Egg white	5.7
Vanilla flavoring	4.4
Almond^a^	85.0

Total	220.2

a Almond was in the form of almond flour (AF, <450 µm) or almond particles (AP, 1700–2000 µm).

### 2.2 Simulated digestion

For the *in vitro* digestion, human mastication was followed by digestion within the dynamic gastric model (DGM) and a static duodenal model. The DGM provides a realistic simulation of the physical and chemical processes within the human stomach, and accurately mimics the transit time and luminal environment (Mandalari et al., 2013, Pitino et al., 2010). This method allows sampling at pre-determined times throughout gastric and duodenal digestion.

#### 2.2.1 Mastication of almond muffins for simulated digestion

A healthy volunteer was recruited by the Human Nutrition Unit at the Institute of Food Research (IFR) for a series of 8 study days (between November 2013 and February 2014), four for each muffin type, AF or AP. Only one volunteer was used to improve reproducibility between replicates. In previous studies, we found that the particle size distribution of masticated almonds is generally consistent across individuals, so this volunteer is likely to be representative of the general population (Grundy, Grassby et al., 2015). The inclusion and exclusion criteria have been reported previously (Grundy, Grassby et al., 2015). The mastication study received NHS ethics committee approval (10/H0717/096) and informed consent was obtained from the participant. The study was conducted in accordance with the ethical standards laid down in the 1964 Declaration of Helsinki and its later amendments. The study was registered at the ISRCTN registry (ISRCTN58438021). On each study day the volunteer was asked to masticate half a muffin (110 g) and custard (40 g) and expectorate each mouthful when they felt the urge to swallow. Each frozen muffin was cut vertically in half; one half was defrosted at 4 °C the day before the study day, the remaining half was kept frozen (-20 °C) until needed. Mastication of the test meals took 3 min 22 s and 6 min 38 s on average for the AF and AP muffins, respectively. During mastication, the volunteer produced 21.8 g ± 4.2 and 35.8 g ± 3.3 of saliva for the AF and AP muffins, respectively.

#### 2.2.2 Gastric digestion

Individual masticated AF (*n* = 3) or AP (*n* = 3) muffin samples (∼180 g each) were fed into the DGM for 63 min in the presence of priming acid (20 ml), whose composition has been reported previously (Pitino et al., 2010). The digestion time was calculated by the *in silico* model associated with the DGM, based on the physico-chemical properties of the meal (Thuenemann, Mandalari, Rich, & Faulks, 2015). The composition of the simulated gastric acid solution has been reported previously (Mandalari et al., 2014). The simulated gastric enzyme solution was prepared by dissolving porcine gastric mucosa pepsin and a gastric lipase analogue from *Rhizopus oryzae* in the above described salt mixture (no acid) at a final concentration of 9000 U mL^−1^ and 60 U mL^−1^ for pepsin and lipase, respectively. A suspension of single-shelled lecithin liposomes, prepared as previously described (Mandalari et al., 2008) was added to the gastric enzyme solution at a final concentration of 0.127 mM. A total of seven samples (35 g) were removed from the DGM at 9 min intervals (63 min total digestion time). The amounts of acid secretions (means ± SD) added during gastric digestion were 28 mL ± 3 and 21 mL ± 2 for AF and AP muffins, respectively. The amounts of gastric enzymes (means ± SD) added during gastric digestion were 28 mL ± 2 and 29 mL ± 4 for AF and AP muffins, respectively. A control run without addition of gastric enzymes was performed for both AF and AP muffins: the amount of acid secretion added during gastric digestion was 34.6 mL for AF and 26.3 mL for AP. Each gastric sample was weighed, its pH recorded and adjusted to 7.0 with NaOH (1 M) to inhibit gastric enzyme activity.

#### 2.2.3 Duodenal digestion

A pooled sample (42 g), obtained from an aliquot (6 g) of each gastric sample, was transferred to a Sterilin plastic tube for duodenal digestion with the addition of simulated bile solution (10.4 mL) and pancreatic enzyme solution (29.2 mL) and incubated at 37 °C under shaking conditions (170 rpm) for 8 h. Aliquots (10 g at 1–6 h, 15 g at 7 and 8 h) were taken every hour during duodenal incubation and replaced with fresh bile (1.2 mL) and pancreatic enzymes (3.5 mL).

Simulated bile was prepared fresh daily. It contained lecithin (6.5 mM), cholesterol (4 mM), sodium taurocholate (12.5 mM), and sodium glycodeoxycholate (12.5 mM) in a solution containing NaCl (146.0 mM), CaCl_2_ (2.6 mM), and KCl (4.8 mM). Pancreatic enzyme solution contained NaCl (125.0 mM), CaCl_2_ (0.6 mM), MgCl_2_ (0.3 mM), and ZnSO_4_·7H_2_O (4.1 μM). Porcine pancreatic lipase (590 U mL^−1^), porcine colipase (3.2 μg mL^−1^), porcine trypsin (11 U mL^−1^), bovine α-chymotrypsin (24 U mL^−1^), and porcine α-amylase (300 U mL^−1^) were added to the pancreatic solution. All samples were immediately frozen and retained for analyses.

#### 2.2.4 Lipid release determination

Almond muffin test meals (AF and AP), post-mastication samples and digesta residues recovered during simulated gastric and duodenal incubation were analyzed for lipid content. The post-mastication samples and digesta residues were centrifuged (3800 × *g*, 15 min) prior to analysis to remove the liquid phase. The pellet was then dried and analyzed. Lipid extraction was performed using a Soxhlet extraction method with *n-*hexane as solvent (Association of American Cereal Chemists [AOAC], 1995). Lipid release was the total lipid in the muffin minus the lipid content of the residue on a dry weight basis.

#### 2.2.5 Microstructural analysis

Microstructural analysis of the almond muffin test meals (AF and AP), post-mastication samples and simulated digestion aliquots was performed as previously reported (Grundy, Grassby et al., 2015). Briefly, aliquots of AP muffin samples were transferred to vials containing the fixative (2.5% v/v glutaraldehyde) for 2 weeks, followed by post-fixing in 2% (w/v) osmium tetroxide. A graded ethanol series was used to dehydrate the samples, before infusion with propylene oxide, and embedding in Spurr resin. Thin (70 nm) sections and semi-thin (1 µm) sections were cut using a Diatome diamond knife (Leica Microsystems Ltd). The thin sections were then examined using a Tecnai T12 transmission electron microscope (FEI Europe) and AMT camera system. Aliquots of AF muffin samples were directly placed on a microscopy slide and stained with Nile red to highlight lipid (Zeiss Axioskop 2 mot plus microscope, excitation at 510–560 nm, emission at 590 nm).

### 2.3 Ileostomy digestion

For the *in vivo* digestion, volunteers with an ileostomy were asked to eat the test meals. Ileal effluent from the volunteers was collected for lipid content measurements along with postprandial blood samples to determine lipemia and glycemia. Despite the major changes occurring to the morphology of the GI tract following an ileostomy, the ileostomy model has been widely used in studies of resistant starch (Champ, Langkilde, Brouns, Kettlitz, & Le Bail-Collet, 2003). Since the majority of nutrient absorption takes place in the upper GI tract, the effluent recovered at the terminal ileum (via the stoma) is considered to be representative of human digestion, although there is some evidence that gastric emptying time and terminal ileum bacterial counts may be higher in ileostomy subjects than in healthy subjects (Booijink et al., 2010, Robertson and Mathers, 2000). Despite these differences, the alternative method of intubation was rejected as being unlikely to give representative samples.

#### 2.3.1 Study design

A single-blind (researcher-blind), randomized, cross-over study design was used where the participants were randomly allocated to receive the AF or AP muffin meal using a computer generated list of random numbers. The randomization, enrolment and allocation of participants were done by the study investigators. The difference in texture between the interventions meant the subjects were not blinded, but samples were coded so those investigators assessing outcomes were blinded. Sample size calculations, using G-Power 3.1.2, were based on 9 participants completing the study at 80% power and an α-level of 0.05 to detect a 235 mmol min L^−1^ in TAG iAUC difference with an SD of differences of 221 mmol min L^−1^ using data from Berry et al. (2008). The primary outcomes were serum TAG concentrations (iAUC) and ileal effluent lipid content; all other outcomes were secondary or exploratory.

#### 2.3.2 Subjects

This study had participant recruitment and screening in common with the study described by Edwards et al., with the aim of recruiting 8–10 healthy participants with ileostomies (Edwards et al., 2015). The participants were eligible if they had undergone a proctocolectomy for ulcerative colitis, lower bowel cancer or pure colonic Crohn’s disease and had normal stoma function for at least one year previously. Other inclusion criteria included: no allergy to almonds or other test meal ingredients; no previous obstruction of the stoma; BMI in the range 20–35 kg m^−2^; no mouth, throat or GI problem (other than ileostomy); total serum cholesterol <7.8 mmol L^−1^; serum triacylglycerol (TAG) <3 mmol L^−1^; plasma glucose <7 mmol L^−1^; and liver function and blood cell counts within prescribed limits. These were assessed using a screening questionnaire and full medical examination. All screening and study visits took place at the Clinical Research Facility at St Thomas’ Hospital, London, UK between November 2012 and April 2013. The stopping guidelines for this study included clinical review of any participant who experienced adverse events (such as blockages), and discussion within the study team as to whether that adverse event posed sufficient risk to future participants to warrant early termination of the study. The study received NHS ethics committee approval (12/LO/1016) and informed consent was obtained from all participants. The study was conducted in accordance with the ethical standards laid down in the 1964 Declaration of Helsinki and its later amendments. The study was registered at the ISRCTN registry (ISRCTN40517475).

#### 2.3.3 Study day

Following screening, participants were asked to attend two separate study days with a gap of at least one week between visits. Prior to each study day, each participant was advised to fast (except water) from 8 pm having consumed a low fat (9.1 g fat), low fiber (5.2 g fiber) evening meal. On the study day, participants changed their stoma bag and were cannulated in a forearm vein. Baseline blood samples were collected before the participants were given the test meal (220 g AF or AP muffin, plus 80 g of custard) for breakfast, which they were asked to consume within 15 min. The nutritional content of the meals as eaten was: 49.4 g lipid, 25.5 g protein, 88.5 g available carbohydrate and 10.1 g dietary fiber. Lunch (low fat yoghurt and a banana) and dinner (6.5 g fat) were provided 4 h and 10 h after breakfast, respectively. Water was freely available throughout the day. The lunch was given to make the procedure acceptable to volunteers and provided minimal lipid (0.7 g) and dietary fiber (2.7 g non starch polysaccharides) and has previously been shown to have no effect on the postprandial lipemic response (Berry et al., 2008). Values for the lunch food items were obtained from UK Food tables (FSA, 2002). All lipid derived from food consumed during each visit, apart from the test meals, was readily available for absorption, and therefore assumed to be absent from the collected effluent.

#### 2.3.4 Blood samples

Blood samples were collected at baseline and at 15, 30, 45, 60, 90, 120, 150, 180 and 240 min after breakfast for analysis of plasma glucose, insulin and C-peptide, and serum TAG and non-esterified fatty acids (NEFA). Additional samples were collected at 5, 6, 7, and 8 h after breakfast for analysis of serum TAG and NEFA. All samples were collected and stored as described by Edwards et al. (2015).

Glucose (glucose oxidase ILTest™ kit), TAG (triglycerides ILTest™ kit) and NEFA (Randox NEFA kit) concentrations were measured using colorimetric assays on an ILab 650 auto-analyzer. Insulin, C-peptide and gut hormones (glucose-dependent insulinotropic polypeptide (GIP), glucagon-like peptide-1 (GLP-1), and polypeptide YY (PYY)) were analyzed according to Filippou, Berry, Baumgartner, Mensink, and Sanders (2014). Cholecystokinin (CCK) was analyzed by the method described in Edwards et al. (2015). Incremental area under the curve (iAUC) values were calculated in Excel 2007 using the trapezoid rule for TAG (480 min), glucose, insulin and C-peptide (120 min).

#### 2.3.5 Analysis of ileal effluent

Effluent samples were collected by the participants, by transferring the contents of their stoma bag to the sample bags at 2 h intervals for 10 h, and then at the participants’ convenience for a further 16 h. Effluent was weighed and frozen at −20 °C immediately on collection, and then transferred to −80 °C within 8 h. Moisture content was measured by freeze-drying the samples (∼50 g). These were then ground up prior to Soxhlet extraction in *n*-hexane to determine lipid content in duplicate. It was assumed that all lipid released from the muffin matrix was digested and absorbed *in vivo*; therefore lipid digestion is equivalent to the total starting lipid in the muffin minus the lipid content of the ileal effluent. Aliquots of the effluent were collected at each time point and prepared for microscopy as described above.

### 2.4 Statistical analysis

Data from the *in vitro* digestion were analyzed using SPSS version 17.0. For all tests, the significance level was set at *P* < 0.05 (2 tailed). All data are expressed as means ± SEM. Repeated-measures ANOVA was used to test for differences in lipid release after mastication, gastric and duodenal digestion with muffin type as a ‘within-sample’ factor. Differences in lipid release between the AF and AP muffin samples were analyzed by Student’s paired *t*-test.

## 3 Results

### 3.1 Total lipid release during mastication and simulated digestion

The cumulative release of lipid, as a percentage of the original lipid present in each muffin, after mastication, *in vitro* gastric and gastric plus duodenal digestion is reported in Table 2. As expected, the total lipid released was significantly (*P* < 0.005) higher for the AF (97.1 ± 1.7%) muffin than the AP muffin (57.6 ± 1.1%). Repeated-measures ANOVA showed that the differences in lipid release between the different stages of digestion were significant (*P* < 0.001). For the AF muffin, an increase in lipid release was observed during the gastric phase compared with that detected in the masticated samples. In accordance with our previous investigation (Mandalari et al., 2014), duodenal digestion produced a significant increase in lipid release over and above that of the gastric phase alone, for both meals. The “blank” runs, that did not include digestive enzymes, had total lipid release values of 16.1% and 2.2% from the AF and AP muffins, respectively.

**Table 2:** Total lipid release (%) from muffins containing almond flour (AF, *n* = 3) or almond particles (AP, *n* = 3) after mastication, *in vitro* gastric and gastric plus duodenal digestion (total digestion time was 9 h). Values are presented as means ± SEM.

	AF	AP
Lipid released after mastication (%)	4.4 ± 0.4	1.9 ± 0.2
Lipid released after gastric digestion (%)	41.6 ± 1.6	5.8 ± 0.1
Lipid released after gastric plus duodenal digestion (%)	**97.1** ± **1.7**	**57.6** ± **1.1**

Recovered lipid (%)	2.9 ± 1.7	42.4 ± 1.1

Total lipid released is calculated relative to the lipid content of the muffin (24 g of lipid).

Repeated-measures ANOVA showed that the differences in lipid release between the different stages of digestion were significant (*P* < 0.001).

Total lipid released was significantly (*P* < 0.005) higher for the AF muffin than the AP muffin.

AF, muffin containing almond macroparticles.

AP, muffin containing almond flour.

### 3.2 Lipid digestion *in vivo*

After the first participant completed both study days without any adverse events, two participants experienced temporary obstruction of the stoma following ingestion of their first test meal, leading to termination of the study on ethical grounds (see CONSORT diagram, Supplementary Fig. 1 in supplementary information). Data is therefore shown for the one completed volunteer only.

**Fig. 1:**
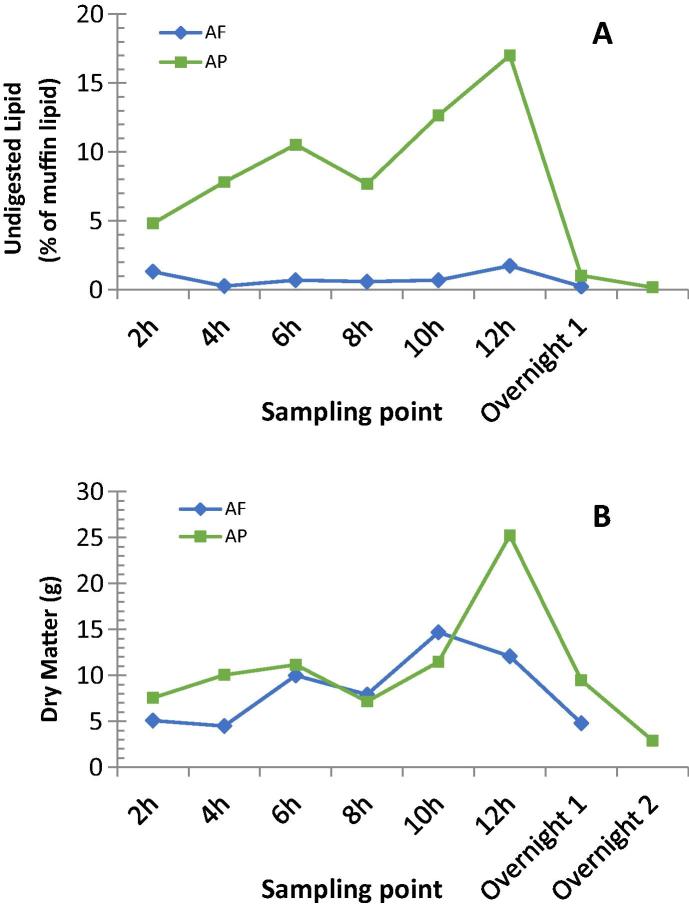
Proportion of undigested lipid in the effluent samples (A) and dry matter content of effluent (B) recovered at each sampling point from the terminal ileum of the ileostomy volunteer (n = 1) for AF (green) and AP (blue). Lipid values are calculated as a percentage of the muffin lipid (48 g). Lipid analysis of duplicate samples. (For interpretation of the references to colour in this figure legend, the reader is referred to the web version of this article.)

The effluent collections were analyzed for dry matter and lipid content, which are presented in Table 3. Results for 0–10 h are reported for comparison to the *in vitro* data, but as almond particles were still being recovered the morning after the study day, 0–24 h data are also shown. The lipid digested after ingestion of AF and AP muffins, over 0–10 h was 96.5% and 56.5%, respectively. The undigested lipid recovered in the effluent at each time point, as a proportion of that ingested in the muffin (48 g), is presented in Fig. 1A, showing clear differences between the test meals. The ileal effluent recovered 2 h after consumption of the AF muffin had the second highest undigested lipid content for that meal (1.3% lipid); which may indicate that 2 h is insufficient time to fully digest the accessible lipid (i.e. mainly from fractured almond cells). For the AP meal, the effluent recovered after 12 h had the highest undigested lipid content (17.0%). The dry matter content of the ileal effluent for each time point is presented in Fig. 1B. Overall, more dry matter was excreted after consumption of the AP muffin compared with the AF muffin. A significant portion of the former was recovered at 12 h, which may indicate that the evening meal pushed through any almond particles remaining in the stomach.

**Table 3:** Characteristics of ileal effluents after ingestion of muffins containing almond flour (AF) or almond particles (AP) over 0–10 h or 0–24 h. (n = 1).

	AF	AP
Total weight, 0–10 h (g)	465.8	423.9
Total dry matter, 0–10 h (g)	42.2	47.4
Total lipid content, 0–10 h (g)	1.7	20.9
Lipid digested, 0–10 h (%)^a^	**96.5**	**56.5**
Recovered lipid, 0–10 h (%)^a^	3.5	43.5

Total weight, 0–24 h (g)	672.7	726.9
Total dry matter, 0–24 h (g)	59.0	85.0
Total lipid content, 0–24 h (g)	2.7	29.6
Lipid digested, 0–24 h (%)^a^	94.4	38.3
Recovered lipid, 0–24 h (%)^a^	5.6	61.7

a Percentage values calculated as a proportion of the lipid content in the almond muffins only (i.e. 48 g of lipid). Lipid content was measured in duplicate.

### 3.3 Effects of mastication and digestion on almond microstructure

During sample processing, almond particles could be easily identified by eye in the effluent obtained from the chewing and digestion of both test meals.

#### 3.3.1 Mastication and *in vitro* digestion

**Fig. 2:**
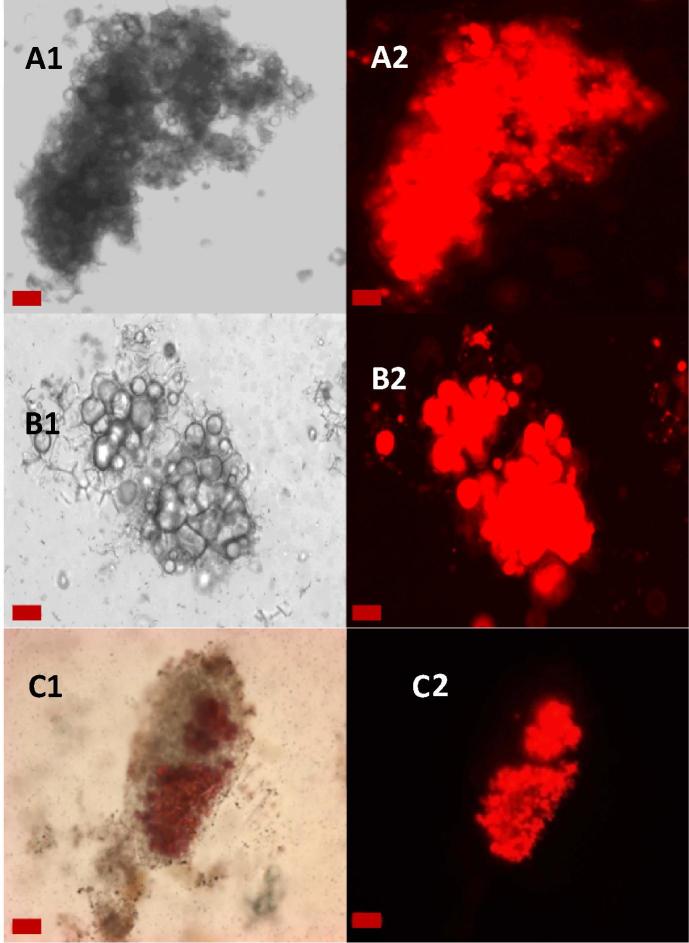
Light micrographs unstained (A1, B1) and stained with Nile red (C1, A2-C2) of almond particles from AF recovered at different stages of digestion: baseline muffin (A), after *in vitro* gastric and duodenal digestion (B), and ileal effluent at 8 h (C) of digestion. Lipids are stained in red with Nile red. Scale bars: A and B = 20 μm; C = 50 µm*.* (For interpretation of the references to colour in this figure legend, the reader is referred to the web version of this article.)

Although the particles were examined at all stages of digestion, changes to the microstructure of the AF particles (comprising the lipid-rich cotyledon cells) were only observed after gastric plus duodenal digestion (Fig. 2A and B). Staining the lipid with Nile red highlighted the loss of lipid from the cells at the periphery of the AF particles. Some lipid remained encapsulated in the cells, in agreement with the biochemical quantification of lipid. Although it is difficult to evaluate quantitatively, the majority of the lipid appeared to have coalesced.

**Fig. 3:**
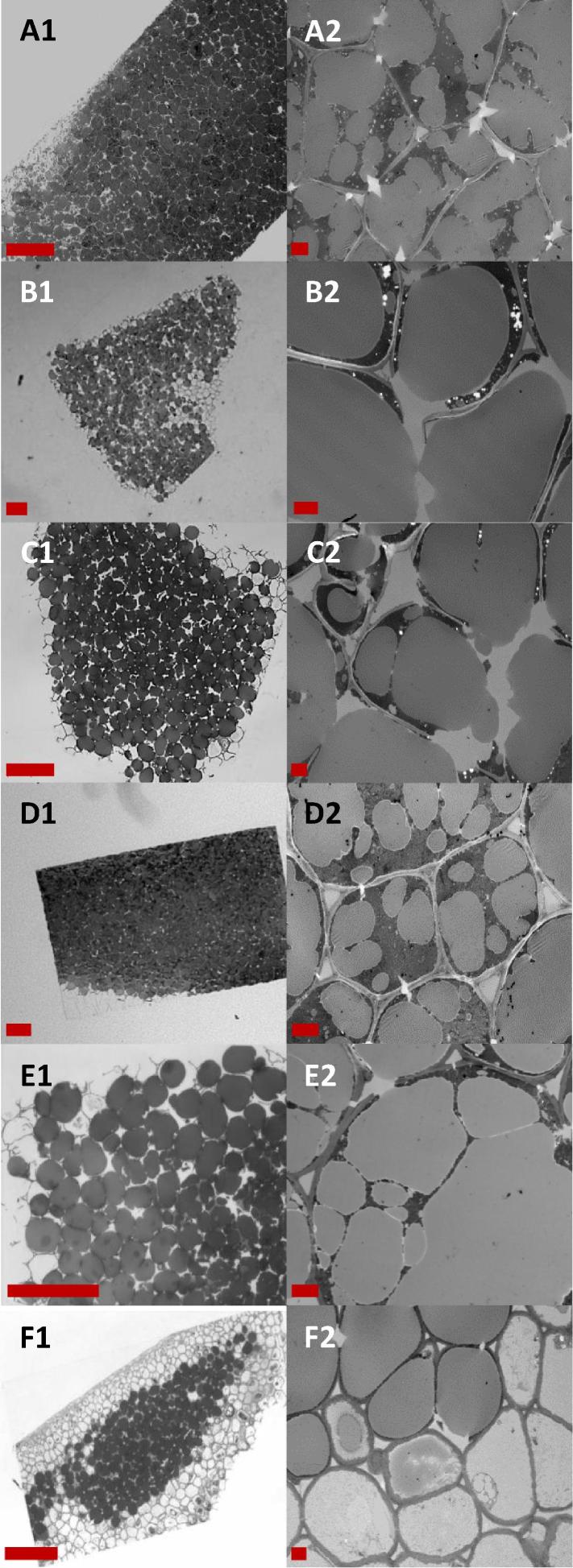
Light micrographs (A1-F1) and TEM micrographs (A2-F2) of sections of almond particles from AP recovered at different stages of digestion: baseline (pre-digested) muffin (A), ileal effluents at 2 h (B), 4 h (C), 8 h (D) of digestion, *in vitro* post gastric and duodenal digestion (E), and ileal effluent at 12 h (F) of digestion. Scale bars: A1 – F1 = 100 μm; A2 – F2 = 5 μm.

These observations were also reflected in the large almond particles where the lipid had been fixed with osmium tetroxide (Fig. 3A and E). The lipid within raw almonds is found in oil bodies (1–3 µm in diameter), but after processing and subsequent digestion they had all coalesced to form large oil droplets (5–35 µm diameter) (Fig. 3A2 and E2). In some parts of the tissue the cell walls had fractured (possibly during baking and/or freezing of the almond muffins), but the cell contents were still largely trapped within the tissue matrix.

#### 3.3.2 *In vivo* digestion

The micrographs of almond particles collected after ingestion of the AF muffin show that there are some particles which still contain encapsulated lipid after 8 h of digestion (Fig. 2C). In fact, there were some particles that contained encapsulated lipid even after 20 h of digestion (data not shown). However, these particles are small (<400 µm) and therefore make up a small proportion of the lipid that was initially present (∼ 3 µg for each 400 µm particle). The structural integrity of the particles appeared largely unchanged throughout the digestion, although some changes, such as particle erosion, cannot be excluded by microscopy. In both the *in vitro* and *in vivo* digestions some fissures are identifiable in the micrographs, and the lipid is clearly bioaccessible where these fissures have compromised the cells.

Micrographs of the larger particles recovered after ingestion of the AP muffin, (Fig. 3B–D and F) showed that undigested lipid remained trapped within the almond particles despite apparent damage to the cell walls. However, progressive loss of lipid from the surface of the large particles (Fig. 3F1) suggests that the cell wall damage made some of the lipid near the surface bioaccessible, and perhaps that the intact cell walls become less efficient barriers to digestion with time. While the lipid loss was never complete, it became more noticeable at the later stages of digestion (>10 h). It was also noted that some swelling of the almond cell walls, at least in cells that had lost their contents, seemed to occur as digestion time increased (Fig. 3F2), in agreement with an earlier study (Mandalari et al., 2008). The microstructural features of particles digested for the same period of time *in vitro* or *in vivo* appeared to be similar to each other (i.e. samples recovered at 8 h).

### 3.4 Postprandial metabolic responses

As results from a single participant are presented, care must be taken when interpreting the data. However, the results add to the limited published data from people with an ileostomy and provide some useful insight into the metabolic responses to the two different forms of almond used in the muffins.

#### 3.4.1 Serum lipid concentrations

**Fig. 4:**
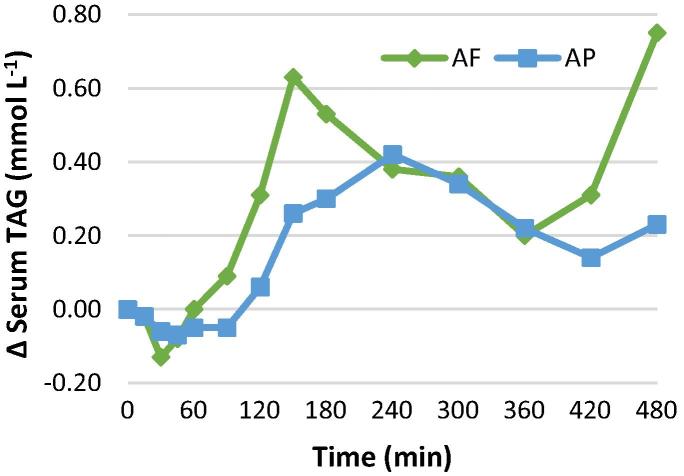
Changes from fasting in serum TAG concentrations in an ileostomy volunteer (n = 1) after the test meals containing 48 g of lipids. AF (green) or AP (blue). (For interpretation of the references to colour in this figure legend, the reader is referred to the web version of this article.)

Changes in postprandial serum TAG concentrations are presented in Fig. 4. The serum TAG iAUC (480 min) was ∼61% higher for AF muffins than AP muffins (148.8 vs 92.3 mmol min L^−1^), with peak serum TAG at 2.5 h for AF (0.63 mmol L^−1^ greater than fasting) and 4 h for AP (0.42 mmol L^−1^ greater than fasting). Serum concentrations of NEFA (Supplementary Fig. 2) following ingestion of AF and AP were lower than the fasting values for the whole 8 h tested.

#### 3.4.2 Plasma glucose, insulin, C-peptide and gut hormone concentrations

The iAUC (0–120 min) for postprandial glucose, insulin and C-peptide responses were 30% (98 vs 140 mmol min L^−1^), 36% (15.0 vs 23.5 nmol min L^−1^) and 30% (112 vs 161 nmol min L^−1^) lower for AF than AP, respectively. The pattern of the postprandial responses for all three of these measures were broadly similar (Supplementary Fig. 2) with a broad peak at 3 h for the AF muffin (2.0 mmol L^−1^, 0.219 nmol L^−1^ and 1.58 nmol L^−1^, above baseline respectively) and a sharp peak at 30 min for the AP muffin (2.8 mmol L^−1^, 0.460 nmol L^−1^ and 2.130 nmol L^−1^, above baseline respectively). Postprandial GIP, GLP-1, CCK and PYY concentrations in response to the two test meals had different patterns of response as shown in Supplementary Fig. 3. The CCK concentrations after ingestion of the AP meal were considerably reduced below fasting levels.

## 4 Discussion

Here we have demonstrated, using *in vitro* and *in vivo* models of digestion, that the bioaccessibility of almond lipid within a complex food matrix is significantly affected by the size of the almond particles in the food. The testing of the same experimental meals in both models was originally performed to allow validation of the *in vitro* model as a good mimic of human digestion under realistic physiological conditions. Although only one participant completed the *in vivo* study, it was still possible to tentatively compare the models in terms of the proportion of lipid released from the matrix and the microstructural changes during digestion, and indeed the results showed very good agreement for both outcomes. The difference in total lipid digestibility between the AF and AP muffin meals obtained with the DGM model (9 h total digestion time) and the ileostomy volunteer (up to 10 h digestion time) was ∼40%. The good agreement between the models could be attributed to our use of an *in vivo* mastication step, as this step is arguably the hardest stage to model *in vitro* (Minekus et al., 2014). The greater recovery of lipids in ileal effluent following consumption of the AP muffin compared with the AF meal is consistent with the results of Cassady, Hollis, Fulford, Considine, and Mattes (2009) who found increased fecal lipid content when samples were chewed fewer times (larger average particle size).

The importance of encapsulation of nutrients within intact cell walls (i.e. a barrier mechanism) has been studied previously under similar conditions (Grundy, Grassby et al., 2015, Mandalari et al., 2008), but without incorporation of almonds into a processed food matrix. An investigation of the postprandial effects of encapsulated vs completely free lipid within a muffin was recently performed in healthy subjects, which showed the importance of lipid bioaccessibility for postprandial lipemia (Berry et al., 2008). The effect of the food matrix on the absorption of artificially encapsulated fish oil has been assessed in ileostomy subjects or volunteers, although in this case the purpose of encapsulation was the prevention of lipid oxidation (Sanguansri et al., 2013). This present study builds on our previous work (Berry et al., 2008, Grundy, Grassby et al., 2015, Mandalari et al., 2008, Mandalari et al., 2014) by measuring lipid bioaccessibility from naturally-encapsulated lipid within a food matrix (muffins), while simultaneously observing the structural changes within the upper GI tract (with a suitable *in vitro* model), and the effect of particle size on postprandial metabolic responses.

The proportion of lipid digested in the DGM was greater for both muffin meals than masticated raw and roasted almonds (∼32% for both almond types) studied previously (Mandalari et al., 2014). This may be because the muffin meals were digested for 9 h with the digestive enzymes replenished at 2 h intervals, whereas the masticated almonds were digested for only 3 h. The digestion time was extended for the current study in order to better reflect the transit time typically experienced by the ileostomy volunteer and also that TAG takes longer to appear in the bloodstream. The form of the digested almonds may have affected the process as well. In the current study the muffin matrix does not appear to have impeded lipid bioaccessibility and digestion. The test muffins consisted of almond particles in a matrix of sucrose, protein and gelatinized starch, which were probably rapidly digested and therefore unlikely to have formed a significant barrier to digestion of the almond lipid. The degree to which the tissue structure remains intact after pre-ingestion processing and mastication, appears to have a strong effect on the rate and extent of the lipid digestion.

It is interesting to note that there was limited lipid digestibility of the AP despite the cell wall damage identified by the microstructural analysis. One would expect the cell wall damage to allow free diffusion of digestive enzymes and lipid through the tissue. However, this exposure of intracellular lipid caused by cell wall rupture as well as possible lipid molecules present at the interface may have been offset by the lack of a continuous aqueous phase, since lipase can only act at the water-lipid interface, facilitated by colipase and bile salts (Grundy et al., 2016). In addition, the coalescence of the lipid may have limited the rate of reaction due to the decreased surface area to volume ratio, although this would apply to both the AF and AP. The lipid coalescence probably happened when the muffins were baked, whereas the damage to almond cell walls may have been caused by mechanical processing of the almonds and/or freezing the almond muffins prior to digestion. The processing method used to prepare the samples for microscopy does not seem to result in cell wall damage or lipid coalescence (Grundy, Grassby et al., 2015).

The recent study by Sanguansri et al. investigated the effect of incorporating microencapsulated omega-3 long-chain polyunsaturated fatty acids (LCPUFA) into a food matrix on lipid release in the upper gastrointestinal tract (Sanguansri et al., 2013). Regardless of whether the oil was supplied as fish oil capsules or as a microencapsulated powder incorporated into a food matrix, specifically orange juice, yoghurt and a cereal bar; only 0.58–0.73% of the total LCPUFA was recovered in the ileal effluent. In the matrices that Sanguansri and colleagues used, the oil was only mixed with the other ingredients to protect the oil during processing, rather than being an integral part of an intact tissue, which explains why in our study the encapsulated lipid in the almond tissue was not fully digested.

The postprandial serum responses of a single subject showed that the AP muffin produced an attenuated lipemic response, compared with the AF meal. Berry et al. reported that muffins containing large particles of almond elicited plasma TAG concentrations that were 74% lower (0–8 h iAUC) than for muffins containing almond oil and defatted almond flour (Berry et al., 2008). The larger reduction in TAG iAUC reported by the Berry group (74%) compared with the result from this study (38%) may be explained perhaps by the greater lipid bioaccessibility of the almond oil (100%) they used relative to that of the almond flour (49%) used in our present study. However, this comparison should be treated with caution due to the differences in participant characteristics (healthy vs ileostomy subjects) and that in the current study data from a single participant is reported. In the present investigation, the lipemic response was also delayed following ingestion of the AP meal compared with the AF meal. This observation is in agreement with Burton-Freeman, Davis, and Schneeman (2004).

Despite having similar carbohydrate contents (88.5 g) the AF muffin produced an attenuated, but prolonged plasma glucose response, which may be due to more bioaccessible lipid being present in the muffin matrix. This additional ‘free’ lipid may firstly reduce the rate of gastric emptying and influence subsequent gut hormone release, as previously reported by our group (Berry, Lapsley, & Ellis, 2009). Secondly, amylose-lipid complexes may be formed while mixing the batter and baking the muffins, particularly in the AF muffins where there was more available lipid. These complexes are hydrolysed more slowly by amylase than amylose alone (Singh, Dartois, & Kaur, 2010).

The main strength of this study is the direct comparison of *in vitro* and *in vivo* digestion of the same test meals with identical compositions, but markedly different levels of lipid bioaccessibility. *In vivo* mastication was used for both digestions, as *in vitro* simulation of the particle size reduction and salivary secretion associated with masticating complex foods *in vivo* is often unsatisfactory (Cassady et al., 2009). As mastication is the main process by which particle size is reduced (and therefore lipid bioaccessibility increased) it was essential to use the same process for both models. The main limitation of this study was the premature termination of the *in vivo* study, resulting in data from only one participant being available. The inherent resistance of these almond structures to digestion seems to make them problematic for some individuals with an ileostomy. We think that the blockages were caused because the matrix was digested from around the almond particles, allowing the hard angular particles to lock together to form a hard plug which the movements within the intestine could not break up. Therefore almonds and similar foods may be limited in their potential use for future ileostomy studies. The data presented from this one ileostomy participant, who did tolerate these meals, have clearly shown that almond cellular integrity has a substantial effect on lipid bioaccessibility in the small intestine. They also provide confirmation of suspected mechanisms behind the limited digestibility of the nutrients in almonds, which will inform future studies. It may be possible to repeat the study in ileostomy volunteers by substantially reducing the almond content of the meals to a level the volunteers eat in their usual diets; however healthy volunteers may be necessary for studies where the primary outcomes dictate that large quantities of nuts be ingested. For future ileostomy studies, we would advise trialing any meals containing significant quantities of nuts prior to the main study.

In conclusion, this study confirmed that decreasing the size of almond particles, and therefore reducing the proportion of intact cells, increased the proportion of lipid digested by *in vitro* and *in vivo* methods. A significant portion of the lipid remained separated from the digestive enzymes by the physical barrier of intact cell walls within the almond particles, even after 12 h of *in vivo* digestion. The proportion of lipid digested seemed to be reflected in the blood lipid, glucose and insulin responses, although the structure and composition of the food matrix also had some influence on these results. The coupling of an *in vivo* mastication step with the *in vitro* model showed good agreement with the *in vivo* modeling of digestion, and in future studies this may provide a cheaper alternative for studying complex food systems.
