# Supplementary material for: In vitro and in vivo modeling of lipid bioaccessibility and digestion from almond muffins: The importance of the cell-wall barrier mechanism
Source: J Funct Foods. 2017 Oct;37:263–71. doi: 10.1016/j.jff.2017.07.046 (PMC5628021; doi:10.1016/j.jff.2017.07.046)
Supplement: Supplementary Figs. 1–3 [file mmc1.docx]

**Supplementary Figure 1. CONSORT diagram.** Flow of participants through the study. Study 1 refers to the lipid bioaccessibility study described in the present work, whereas Study 2 refers to a parallel study on starch bioaccessibility that was reported elsewhere (Edwards et. al.; *Am. J. Clin. Nutr.* 2015, 102:791-800). Study participants were recruited and screened through a common process, then allocated to take part in Study 1 and/or 2, depending on eligibility and availability (Study 1 and 2 had different inclusion and exclusion criteria).

**Supplementary Figure 2. Serum NEFA (A), plasma glucose (B), insulin (C) and C-peptide (D) concentrations in an ileostomy volunteer (n=1) after consumption of the test muffin meals containing 48 g of lipids from AF (green) or AP (blue) muffins.**

**Supplementary Figure 3. Plasma GIP (A), GLP-1 (B), CCK (C)and PYY (D) concentrations in an ileostomy volunteer (n=1) after consumption of the test muffin meals containing 48 g of lipids from AF (green) or AP (blue) muffins.**

**Supplementary Figure 1.**

Enrollment

**Assessed for eligibility (n = 17)**

Analysis

Follow-Up

**Visit 1:**

♦ Received allocated intervention (n = 1)

♦ Discontinued intervention due to abdominal discomfort (n = 2)

**Analysed (n = 1)**
♦ Excluded from analysis (n = 0)

**Visit 2:**

♦ Received allocated intervention (n = 1)

**Allocated to Study 1 (n = 8)**

♦ Received allocated intervention (n = 3)

♦ Not allocated to Study 1 (n = 5)

- Abdominal discomfort from Study 2 (n = 1)
- Termination of the study on ethical grounds (n = 4)

Allocation (n = 13)

**Excluded (n= 4)**

♦  Not meeting inclusion criteria for either study (n = 4)

**Allocated to Study 2* (n = 11)**

♦ Not allocated to Study 2 due to abdominal discomfort from Study 1 (n = 2)

* Data from Study 2 reported in Edwards et al., 2015

**Supplementary Figure 2.**

**Supplementary Figure 3.**
